# Mycobacterium tuberculosis combine with EBV infection in severe adult meningoencephalitis: a rare case reports and literature review

**DOI:** 10.3389/fcimb.2024.1361119

**Published:** 2024-10-14

**Authors:** Jian Wang, Mengjiao Li, Junchi Zhu, Lijuan Cheng, Ping Kong

**Affiliations:** Department of Neurology, Affiliated Aerospace Hospital of Zunyi Medical University, Zunyi, Guizhou, China

**Keywords:** tuberculous meningitis, Epstein-Barr virus, meningoencephalitis, metagenomic next-generation sequencing, case report

## Abstract

**Background:**

Tuberculous meningitis (TBM) with adults Epstein-Barr (EB) virus encephalitis is a very rare infectious disease, with a high mortality and disability. Metagenomic next-generation sequencing (mNGS) of cerebrospinal fluid (CSF) is highly diagnostic. We report on a case of severe meningoencephalitis caused by co-infection with mycobacterium tuberculosis and EB virus. Brain MRI indicated a parenchyma lesion in the brain. mNGS of CSF indicated Mycobacterium tuberculosis and EB virus amplification, positive serum EB virus IgG antibodies, and improved symptoms after anti-tuberculosis and antiviral treatment. A re-examination of the brain MRI revealed that the significantly absorption of the lesions.

**Case report:**

A 49-year-old male patient presented with a chief complaint of headache and fever with consciousness disturbance. The brain magnetic resonance imaging showed a lesions in the right parenchymal brain with uneven enhancement, accompanied by significantly increased intracranial pressure, elevated CSF cell count and protein levels, as well as notably decreased glucose and chloride levels. mNGS of CSF showed the coexistence of Mycobacterium tuberculosis and EBV. The patient was diagnosed as TBM with EBV encephalitis. The patient’s symptoms gradually improved with the active administration of anti-tuberculosis combined with antiviral agents, the use of hormones to reduce inflammatory reaction, dehydration to lower intracranial pressure, and intrathecal injection. Subsequent follow-up brain magnetic resonance imaging indicated significant absorption of the lesions, along with a marked decrease in CSF count and protein levels, as well as obvious increase in glucose and chloride levels.

**Conclusion:**

TBM associated with adult EBV encephalitis is extremely rare. The disease’s early stages are severe and have a high fatality rate. A prompt and accurate diagnosis is particularly important. NGS of CSF is of great value for early diagnosis.

## Introduction

Tuberculous meningitis (TBM) is a non-suppurative inflammatory disease of the central nervous system caused by Mycobacterium tuberculosis, with a high mortality and disability rate (Ahlawat et al., 2020). adults Epstein-Barr virus(EBV) encephalitis is a serious infectious disorder of the central nervous system, predominantly observed in children and relatively rare in adults. It poses a perilous threat, with an alarmingly high fatality rate (Baldwin and Cummings, 2018). TBM co-infection with EBV is extremely rare. We hereby reported an analysis and summary of a case of rare severe TBM simultaneously infected with EBV in our hospital.

## Case report

We reported a case of a 49-year-old man complaining of headache and fever with impaired consciousness. According to the description of the patient’s family: on October 21,2023, the patient felt headache characterized by a diffuse, persistent pain throughout the entire head. The headache was accompanied by severe nausea and vomiting, which did not alleviate the pain. As well, the administration of oral analgesics failed to provide any relief for the headache. On October 23,2023, the patient began to experience a fever, with the highest body temperature ranging from 39°C to 41°C. Upon self-administration of antipyretic medication ibuprofen, the temperature briefly returned to normal, only to rise again after a few hours. He was diagnosed with purulent meningitis after examination at a local hospital. Treatment included the administration of mannitol dehydration to reduce intracranial pressure, cephalosporin for anti-inflammatory, and fever reduction. However, the specific treatment plan was unknown. The patients complained of persistent headache without obvious alleviation, still frequent vomiting, and continuous fever with temperature fluctuations ranging from 39°C to 41°C. The condition progressively worsened, and the patient exhibited abnormal behavior characterized by incoherent speech and restlessness. On October 28, 2023, the patient presented with impaired consciousness, unresponsive, and in a state of coma. Due to continuous deterioration, he was immediately transferred to our hospital. According to the family, the patient had a history of silicosis for more than 10 years, with occasional difficulty breathing. No special personal or family history was found.

The physical examination revealed a temperature of 40.4°C, a pulse rate of 128 times/min, a respiratory rate of 28 times/min, a blood oxygen saturation of 80% without oxygen supplementation, and a blood pressure of 132/87 mmHg. The patient appeared to be in a state of shallow coma, with diminished sound and moist rales in both lungs. Further examination of the internal medicine was inconclusive. In the neurological examination, the patient was in a state of mild coma, with bilateral pupil round and dilated, measuring approximately 2.5 mm in diameter. He exhibited sensitive response to light stimuli, and displayed a sign of distress during pressure frame reflex. The patient was uncooperative during the cranial nerve examination. Avoidance movement of limbs were observed in response to painful stimuli, and symmetrical limb tendon reflexes were present, with neck rigidity 3 fingers and Kernig sign positive. No pathological signs were elicited.

**Figure 1:**
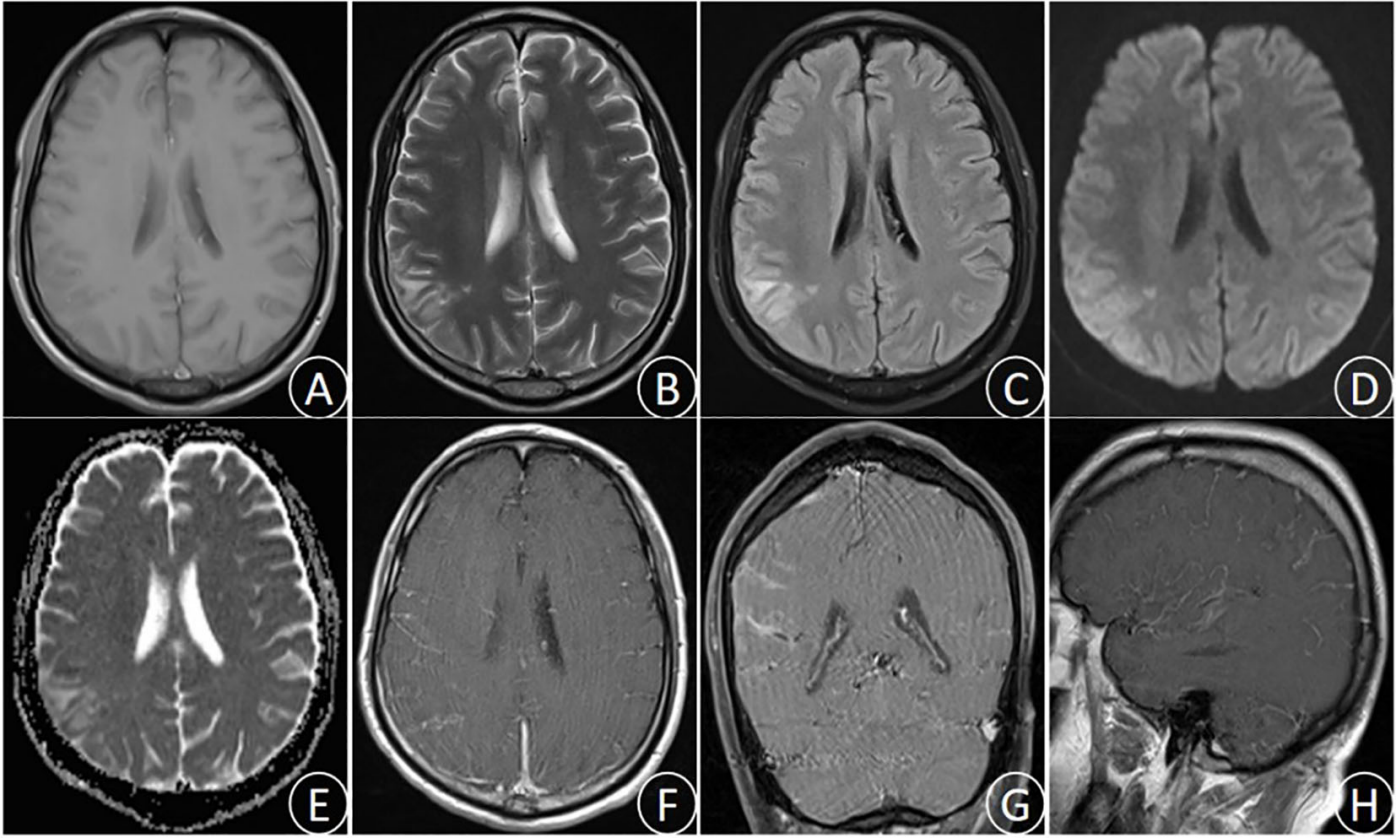
(October 28th,2023) MRI images showed the right parietal lobe long T1 signal **(A)**, long T2 signal **(B)**, slightly increased FLAIR signal **(C)**, high DWI signal **(D)**, low ADC signal **(E)**, and sulcus gyrus edema. Enhanced MRI images **(F–H)** showed uneven enhancement in the right parietal lobe.

After admission to our department, the patient received active treatment included endotracheal intubation for assisted ventilator, continuous oxygen inhalation, electroencephalographic(ECG) monitoring of vital signs, administration of mannitol dehydration to reduce intracranial pressure, cephalosporin for anti-infection, and antipyretic. Emergency brain magnetic resonance imaging (MRI) examination showed long T1 and long T2 signal in the right parietal lobe, slightly increased FLAIR signal, high DWI signal, low ADC signal, accompanied by sulcus and gyrus edema. Enhanced scan indicated mild enhancement at the center of the lesion (**Figure 1**). Lumbar puncture cerebrospinal fluid (CSF) examination demonstrated the increased brain pressure (> 400 mmH2O), elevated CSF cell count and protein levels, and significant decrease in glucose and chloride levels (**Table 1**). The blood routine test indicated a white blood cell count of 12.51 × 10^9^/L, with nucleated cell percentage of 92.7% and a lymphocyte percentage of 2.5%. The erythrocyte sedimentation rate was 74 mm/h, and C-reactive protein (CRP) level was 132.4 μg/ml. Thereafter, a combination of isoniazid, rifampicin, pyrazinamide and ethambutol was administrated for diagnostic anti-tuberculosis (Isoniazid 0.3 qd, Rifampicin 0.45 qd, Ethambutol 0.75 qd, Pyrazinamide 0.5 tid) treatment, and dexamethasone(15mg/daily ivgtt for 2 weeks) was prescribed to reduced inflammation and edema.

**Table 1:** CSF of laboratory results.

CSF	date	CSF pressure (mmH_2_O)	WBC (×10^6^/L)	Nucleated cell (×10^6^/L)	Glucose (mmol/L)	Chloride (mmol/L)	Protein (mg/L)	ADA (U/L)
1	October 28th, 2023	> 400	450	330	0.21	103.25	2380 mg/L	8.99
2	November 1th, 2023	> 400	300	285	0.75	109.53	2160 mg/L	7.94
3	November 1th, 2023	330	260	250	0.83	108.04	1760 mg/L	6.44
4	November 6th, 2023	280	133	120	1.44	109.86	1240 mg/L	5.36
5	November 13th, 2023	220	101	99	1.71	114.01	1030 mg/L	4.78
6	November 20th, 2023	180	90	60	2.12	116.12	630 mg/L	2.17
7	November 30th, 2023	140	75	70	2.08	118.01	580 mg/L	1.46
8	December 5th, 2023	120	42	20	2.38	121.40	490 mg/L	0.49

CSF, cerebrospinal fluid; WBC, white blood cell; ADA, adenosine deaminase.

**Figure 2:**
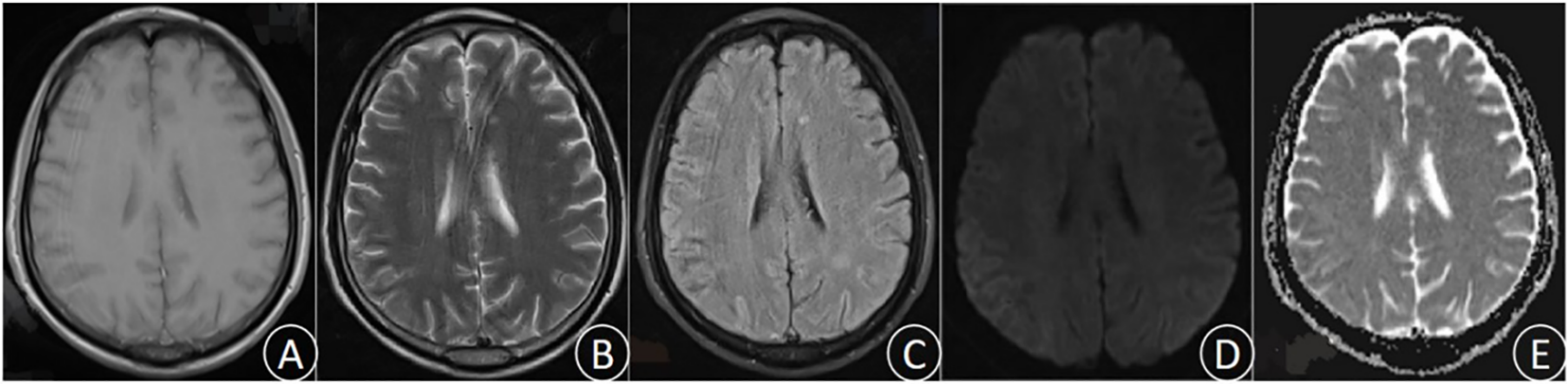
(December 6th, 2023) MRI was performed after treatment. T1 **(A)**, T2 **(B)**, FLAIR **(C)**, DWI **(D)**, and ADC **(E)**.The lesions were significantly improved and the edema was notably alleviated.

After 3 days of continuous treatment, the patient still experienced recurrent high fever and continuous coma. The video electroencephalogram indicated diffuse slow wave distribution. Further CSF and serum autoimmune encephalitis antibodies detection demonstrated the negative of MOG, GFAP, and AQP 4 antibodies. However, the serum EBV-IgG antibody was positive, whereas the tuberculosis antibody was negative. In addition, the repeated CSF antacid staining, cryptococcal ink staining, and cryptococcal capsular polysaccharide antigen test were negative. Blood bacterial cultures were performed multiple times and all yielded negative. The results of CSF metagenomic next-generation sequencing (mNGS) suggested the presence of Mycobacterium tuberculosis (the nucleic acid standardized count was 108) and EBV(the nucleic acid standardized count was 126). Thus, the patient was diagnosed with TBM combine with adult EBV encephalitis, and immediately received acyclovir antiviral (dose 0.8g/day) treatment and intrathecal injection. After 3 days of active treatment, the patient’s consciousness gradually recovered, and the peak body temperature dropped to 38°C. One week later, the endotracheal tube was removed successfully, and the patient was weaned off the ventilator, maintaining a blood oxygen saturation of over 90% under oxygen inhalation. After 10 days of treatment, the patient’s temperature returned to normal, and the headache significantly relieved, with no obvious nausea and vomiting. After 1 month of treatment, the patient had no fever, headache, nausea, and vomiting. His consciousness was clear, with normal sleep and appetite, and normal urination and bowel movements. Follow-up craniocerebral MRI suggested the significant improvement in the right parietal lesion compared to admission (**Figure 2**). Repeat CSF examination suggested the normal pressure, significantly decreased CSF cell count and protein levels, and notably increased glucose and chloride levels.

## Discussion

The patient initially presented with a headache accompanied by a fever, which rapidly progressed to a coma. The clinical diagnosis of TBM was supported by CSF test results. Despite the administration of anti-tuberculosis medication and hormone therapy to reduce inflammatory response, along with alternative treatments, the patient remained in a comatose state with persistent elevated fever, indicating a worsening condition. mNGS of the CSF revealed concurrent infection of Mycobacterium tuberculosis and EBV. Immediate initiation of acyclovir antiviral therapy was administered. Following combined anti-tuberculosis and antiviral treatment, the patient’s consciousness improved, headache subsided, body temperature normalized, and disease progression was gradually controlled. This observation suggests that the patient was co-infected with both Mycobacterium tuberculosis and EBV rather than solely infected with Mycobacterium tuberculosis alone. After 37 days of anti-tuberculosis and antiviral therapy, complete resolution of clinical symptoms was achieved in the patient. Follow-up CSF examination demonstrated a significant decrease in cell counts and protein levels as well as a notable increase in glucose and chloride ion levels. Furthermore, brain MRI revealed evident absorption of the lesion located in the right parietal lobe.

TBM is a severe infectious disease of the central nervous system. It is the most destructive form of tuberculosis, accounting for approximately 5% - 10% of all extrapulmonary tuberculosis, and has a high mortality and disability rate (Donald, 2016; Daniel et al., 2019). The incidence of TBM varies greatly by region, and is influenced by the factors such as overall incidence of TB, age structure, and HIV seropositivity rate (van Crevel et al., 2016; Donovan et al., 2019). A large study including 57,217 cases of extrapulmonary TB showed that TBM constituted approximately 6% of all extrapulmonary TB cases (Donovan et al., 2019). The global burden of TBM is estimated to be at least over 100,000 new cases per year (Gomes et al., 2014). Study of the pathogenesis of TBM is hindered by the lack of experimental models that can reproduce all the characteristics of human disease. Currently, it is believed that the onset of TBM begins with respiratory tract infection, where Mycobacterium tuberculosis spreads to the central nervous system through the bloodstream, and grows and reproduces in individuals with compromised or defective immune function, eventually leading to clinical manifestation of the disease in patients (Meyer et al., 2006). TBM has a high mortality and disability rate, and its mortality soars to approximately 50% in patients coinfected with HIV (Prasad et al., 2016). With diversified clinical symptoms of TBM, non-specific CSF examination, insensitive Mycobacterium tuberculosis culture technique and long culture cycle, the diagnosis of TBM is extremely challenging, often resulting in delayed treatment, and extremely unfavorable prognosis for patients (Riste et al; Van and Farrar, 2013). In recent years, CSF metagenomic next-generation sequencing is a relatively accurate and rapid diagnostic method for TBM, widely used in clinical practice. metagenomic next-generation sequencing (mNGS) is a revolutionary technology that has emerged in recent years and can be used to conduct high-throughput sequencing of microbial nucleic acid in clinical samples and identify pathogens through comparison and analysis with standard sequences in the database. This technique has played an increasingly important clinical role in the diagnosis of CNS infections (Xiang et al., 2023).

EBV, also known as human herpesvirus type 4 (HHV-4), is a B-lymphotropic double-stranded DNA virus identified by Epstein Barr in 1964 (Johnson and Mims, 1968). Human are the only host of EBV, with children being more susceptible to infection. In adults, most EBV infection are asymptomatic, but in rare cases, clinical symptoms may manifest as EBV encephalitis, occurring in approximately 0.4% - 7.5% of cases (Abolhassani et al., 2016; Johnson et al., 2022). EBV primarily spreads through saliva, colonizing within the epithelium of the human body, and then infects B lymphocytes and enters the bloodstream, causing systemic infection (Kennedy, 2020). EBV encephalitis is clinically characterized by fever, headache, and meningeal irritation, and it lacks specificity when compared with other viral encephalitis (Piantadosi and Solomon, 2022; Mendoza et al., 2023). The pathogenesis of EBV encephalitis is currently believed to be related to direct virus invasion, immune induction after infection, and reactivation of immune function when suppressed after virus latency (Sanyal et al., 1991; Piret and Boivin, 2020). Clinical diagnosis of EBV encephalitis mainly depends on serum or CSF EBV-DNA detection and EBV-IgG antibody detection (Steiner and Benninger, 2013; Tselis, 2014). In this case, the patient tested positive EBV-IgG antibody in serum, and the mNGS of the CSF suggested EBV amplification, consistent with the diagnosis of EBV encephalitis.

Silicosis is a pneumoconiosis caused by the long-term inhalation of inorganic dust with high concentrations of free crystalline silica(SiO_2_),this interstitial lung disease is characterized by inflammation, formation of silicotic nodules, and fibrosis, which are progressive and irreversible (Handra et al., 2023; Ing and Kho, 2024). Alveolar macrophages, alveolar epithelial cells, and fibroblasts are involved in the complex pathogenesis of silicosis (Mayeux et al., 2018; Walters and Shukla, 2021). Studies investigating the pathogenesis of silicosis have focused on the roles of alveolar macrophages and alveolar epithelial cells, which secrete pro-inflammatory and profibrotic mediators secondary to exposure to silica, perpetuating the vicious cycle of tissue destruction (Martínez-López et al., 2023).

EBV infection is more common in children. In adults, when immune function is low, EBV lurking in the body can be activated and migrate, causing serious infections and various neurological complications (Tyler, 2018). Studies have demonstrated that EBV has the ability to directly invade neurons, as well as infect vascular endothelial cells and B lymphocytes within the intracranial region,this invasion leads to neurotoxicity through the release of inflammatory factors and viral proteins, ultimately resulting in a range of neurological disorders (Whitley and Gnann, 2002). However, the precise mechanism underlying EBV-mediated central nervous system injury remains incompletely elucidated (Liu et al., 2023).

Considering that the patient in this case had a clear silicosis in the past, we speculated that when the patient’s immune function was low, it might directly lead to the infection of mycobacterium tuberculosis and EBV, or the patient was complicated with hidden tuberculosis, and the migration and activation of mycobacterium tuberculosis and EBV were stimulated after the immune function was reduced, thus inducing a series of inflammatory reactions of the nervous system.

## Conclusion

TBM in combination with EBV-infected adult meningoencephalitis is extremely rare clinically. Its early clinical symptoms are atypical and the condition is critical, with an severely elevated fatality rate. For such cases, timely and accurate clinical judgment, selection of appropriate tests, and prompt early diagnosis and appropriate treatment are critical in saving patients’ lives and reducing mortality and disability rates. NGS of CSF is of great value for early diagnosis.

## Data Availability

The original contributions presented in the study are included in the article/supplementary material. Further inquiries can be directed to the corresponding author.
